# Allogeneic hematopoietic stem cell transplantation should be in preference to conventional chemotherapy as post-remission treatment for adults with lymphoblastic lymphoma

**DOI:** 10.1038/s41409-018-0184-7

**Published:** 2018-04-30

**Authors:** Luxin Yang, Yamin Tan, Jimin Shi, Yanmin Zhao, Yuanyuan Zhu, Yongxian Hu, Wenjue Pan, Yishan Ye, Jingsong He, Weiyan Zheng, Jie Sun, Zhen Cai, He Huang, Yi Luo

**Affiliations:** Bone Marrow Transplantation Center, The First Affiliated Hospital, School of Medicine, Zhejiang University, Hangzhou, People’s Republic of China

**Keywords:** Non-hodgkin lymphoma, Stem-cell research

Lymphoblastic lymphoma (LBL) is a rare and aggressive disease, accounting for less than 2% of non-Hodgkin’s lymphoma (NHL) [1, 2]. Unlike lymphoblastic leukemia (ALL), LBL occurs mainly in young males and is clinically characterized by mass in lymph nodes and extranodal organs with no or minimal evidence of bone marrow involvement (<20%). The response rates have been greatly improved with intensive chemotherapy protocols used for NHL or ALL. However, because of high risk of relapse [3], long-term overall survival (OS) of LBL remains unsatisfactory, with a 5-year OS of 32% and event-free survival of 22% using NHL protocols [4]. It has been reported that allogeneic stem cell transplantation (allo-SCT) achieved lower relapse rates and improved survival in patients with recurrent or refractory disease and those at high risk of resistance or relapse [4, 5]. However, the timing of allo-SCT and which patients may benefit from the treatment are under debate.

We retrospectively analyzed 57 consecutive patients with LBL diagnosed between January 2006 and December 2016 in our institution. Most patients were young males, with 88% of patients having a T-cell immunophenotype. Mediastinal mass was the most common clinical presentation (53%, *n* = 30). Medullary involvement was observed in 29 (51%) patients, while pleural or pericardial involvement was noted in 11 (19%) patients. As a result, 88% of the patients presented with an advanced disseminated disease (III/IV stage). Nearly half of the patients had high-intermediate and high international prognosis index (IPI) (44%, *n* = 25). More details about clinical characteristics were listed in Supplementary Table 1. Induction chemotherapies mainly consisted of the hyper-CVAD regimen (54%, *n* = 31) and the CHOP and CHOP-based regimens (31%, *n* = 18) (Supplementary Table 2). After four courses of induction chemotherapy, 43 patients responded, with an overall response rate of 75% (complete remission (CR): 54%; partial remission (PR): 21%). Local radiotherapy was provided to patients with residual tumor in the mediastinum (*n* = 4) or neck (*n* = 1) after induction chemotherapy with a dose ranging from 20 to 40 Gray. All the 43 responders were completely ambulatory (Eastern Cooperative Oncology Group performance status 0 or 1) after induction chemotherapy. The decision of whether performing a transplantation or chemotherapy after remission was made according to the availability of suitable donor and patient’s willingness. Given the aggressiveness of LBL and GVL effect associated with allogeneic grafts, allogeneic hematopoietic stem cell transplantation (allo-HSCT) was considered prior to auto-HSCT in our center. For patients who aimed to allo-HSCT, a matched sibling donor (MSD) was first preferred, then a matched unrelated donor (MUD), a haploidentical-related donor (HRD) was selected when neither MSD or MUD was available. Conditioning regimen and graft-versus-host disease (GVHD) prophylaxis were carried out as described previously [6]. Responders who did not have a suitable donor or the will to undergo transplantation continued with the effective chemotherapy they had for induction. Those who failed to achieve at least a PR received salvage intensified chemotherapy and were excluded from further analysis. OS and progression-free survival (PFS) were estimated using the Kaplan–Meier method. The Cox proportional hazards regression model was used for multivariate analysis to compare factors with a *p-*value ≤ 0.2 in the univariate analysis. Cumulative incidences of relapse and non-relapse mortality (NRM) were calculated by the competing risk method [7]. A two-sided *p*-value < 0.05 was considered statistically significant.

Twenty patients underwent first-line SCT (17 allo-HSCT and 3 auto-HSCT), while 22 patients continued with chemotherapy. The median interval from the completion of induction chemotherapy to allo-HSCT was 3.8 (range 1.0–8.4) months, one patient was excluded from the chemotherapy group because of the short remission duration of 3.6 months. The distribution of clinical characteristics was well balanced between the two groups (Table 1). The median cycles of chemotherapy offered in chemotherapy group was 7 (range 4–10 cycles). The maintenance therapy included oral 6-mercaptopurine and methotrexate for 2 years in three patients, whereas the other seven patients were observed without additional treatment. Summary of the treatments and responses are displayed in Supplementary Fig. 1. With a median follow-up of 27.2 (range 6.2–91.8) months for surviving patients, the 2-year OS and PFS for all responders were 56 and 35%, respectively (Supplementary Fig. 2). The 2-year PFS was 51% (95% CI, 25–77%) for the allo-HSCT group, comparing with 31% (95% CI, 11–53%) for the chemotherapy group (*p* = 0.034) (Fig. 1a). Meanwhile, the corresponding figures for OS were 58% (95% CI, 32–84%) and 48% (95% CI, 25–71%), respectively (*p* = 0.198) (Fig. 1b). The 2-year cumulative incidence of relapse and NRM for the two groups were 14% vs. 47% (*p* < 0.001), 28% vs. 5% (*p* = 0.061), respectively (Fig. 1c, d). The causes for death after allo-HSCT was infections (*n* = 3), disease progression (*n* = 2), chronic GVHD (cGVHD) (*n* = 1), and secondary graft failure (*n* = 1). A total of 13 deaths occurred in the chemotherapy group (relapsed/progressive disease *n* = 10, infection *n* = 2, osteofascial compartment syndrome *n* = 1). Unfortunately, all the three patients who autografted relapsed and died. Of the 17 patients who underwent allo-HSCT, 16 patients engrafted successfully. Two patients developed grade-III acute GVHD (aGVHD) and two developed severe cGVHD.

**Table 1:** Characteristics of patients in different post-remission therapy groups

Variable	Total	Allo-HSCT group	Chemotherapy group	*p*
*N*	39	17	22	
Gender (%)				0.494
Male	27(69)	13(76)	14(64)	
Female	12(31)	4(24)	8(36)	
Age years median (range)	26(15–61)	26(15–52)	28(15–61)	0.821
Immunophenotye (%)				1.000
T	34(87)	15(88)	19(86)	
B	5(13)	2(12)	3(14)	
Ann Arbor stage (%)				0.449
I	2(5)	1(6)	1(5)	
II	1(3)	–	1(5)	
III	7(18)	3(18)	4(20)	
IV	27(69)	13(76)	14(70)	
N/A	2(5)	–	2(10)	
B symptoms (%)				0.647
Absent	23(59)	9(53)	14(63)	
Present	13(33)	7(41)	6(27)	
N/A	3(8)	1(6)	2(10)	
IPI Index (%)				0.235
0 or 1	12(31)	6(36)	6(27)	
2	5(13)	1(6)	4(18)	
3	10(26)	5(29)	5(23)	
4 or 5	9(23)	5(29)	4(18)	
N/A	3(8)	–	3(14)	
ECOG-PS (%)				0.620
0	1(3)	1(6)	–	
1	15(38)	6(35)	9(40)	
2	21(54)	9(53)	12(55)	
3	2(5)	1(6)	1(5)	
Serum LDH level > normal (%)				0.394
Yes	18(46)	9(53)	9(41)	
No	16(41)	5(29)	11(50)	
N/A	5(13)	3(18)	2(9)	
Medullary involvement (%)				0.478
Yes	22(56)	9(53)	13(59)	
No	16(41)	8(47)	8(36)	
N/A	1(3)	–	1(5)	
Medullary involvement (%)				0.917
≤5%	29(74)	13(76)	16(73)	
>5%	8(21)	3(18)	5(23)	
N/A	2(5)	1(6)	1(4)	
Mediastinal involvement (%)				0.367
Yes	21(54)	8(47)	13(59)	
No	17(44)	9(53)	8(36)	
N/A	1(2)	–	1(5)	
Pleural/pericardial effusion (%)				0.528
Yes	8(21)	4(24)	4(18)	
No	30(77)	13(76)	17(77)	
N/A	1(2)	–	1(5)	
Number of extranodal sites (%)				0.215
0	7(18)	3(18)	4(18)	
1	17(44)	6(35)	11(50)	
≥2	13(33)	8(47)	5(23)	
N/A	2(5)	–	2(9)	
Induction chemotherapy				0.347
Hyper-CVAD A/B	23(59)	10(59)	13(59)	
CHOP/CHOP like	12(31)	4(24)	8(36)	
Others	4(10)	3(17)	1(5)	
Disease status				0.752
CR	28(72)	13(76)	15(68)	
PR	11(28)	4(24)	7(32)	
Radiation therapy				1.000
Yes	4(10)	2(12)	2(10)	
No	35(90)	15(88)	18(90)	
Donor type				
MSD	–	7(40)	–	
MUD	–	5(30)	–	
HRD	–	5(30)	–	

*ECOG-PS* Eastern Cooperative Oncology Group Performance Status, *LDH* lactate dehydrogenase, *IPI* international prognosis index, *N/A* not available, *CR* complete remission, *PR* partial remission, *Hyper-CVAD A* cyclophosphamide, vincristine, doxorubicin, and dexamethasone, *Hyper-CVAD B* methotrexate, cytarabine, *CHOP* cyclophosphamide, doxorubicin, vincristine, prednisolone, *MSD* matched sibling donor, *MUD* matched unrelated donor, *HRD* haploidentical-related donor

**Fig. 1:**
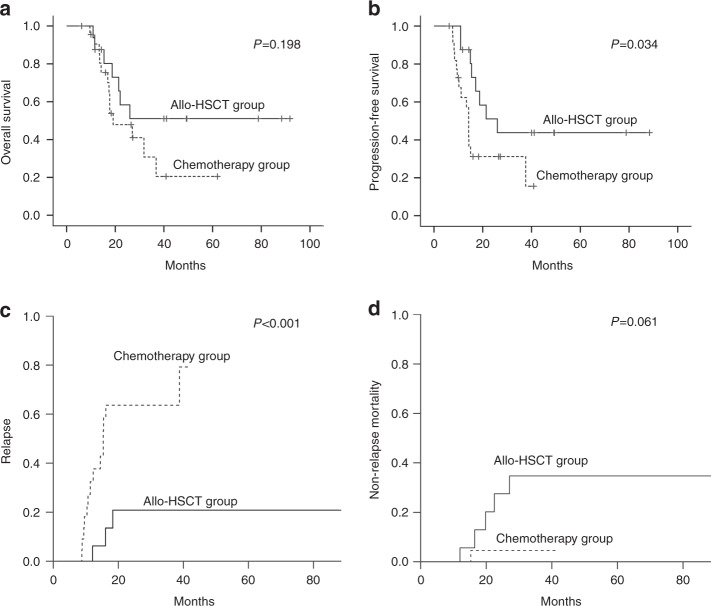
Overall survival (**a**), progression-free survival (**b**), cumulative incidence of relapse (**c**), and non-relapse mortality (**d**) for patients in Allo-HSCT group and chemotherapy group. Allo-HSCT allogeneic hematopoietic stem cell transplantation

In univariate analyses, more extranodal involvements (*p* = 0.01) and poor Eastern Cooperative Oncology Group performance status (ECOG-PS>2) at diagnosis were associated with worse OS (*p* < 0.001). ECOG-PS>2 at diagnosis (*p* = 0.002) and advanced Ann Arbor stage (*p* = 0.053) significantly affected PFS negatively. When entered into multivariate analysis of PFS, only baseline ECOG-PS>2 (*p* = 0.009; HR: 8.158; 95% CI: 1.6794–39.636) achieved statistical significance. Worse OS was predicted by ECOG-PS>2 (*p* = 0.001; HR: 75.496; 95% CI: 6.536–872.080) and radiotherapy (*p* = 0.017; HR: 4.725; 95% CI: 1.325–16.850) (data not shown).

To the best of our knowledge, this is the largest study that compare allo-HSCT with conventional chemotherapy for unselected adults with LBL in first remission. The 2-year PFS was 51% in allo-HSCT group which was significantly superior to that of chemotherapy group (*p* = 0.034). There was a tendency for longer OS in the allo-SCT group but it failed to reach statistical significance, on account of small number of patients and short follow-up. Noteworthily, allo-SCT group was associated with significantly less relapses and a comparable NRM. Allo-HSCT was given to unselected patients regardless of risk stratification and performance status and 24% of patients underwent transplant in PR, perhaps accounting for lower survival rates compared with that of historical studies [8–10]. Even better outcomes were achieved by intensive ALL-type chemotherapy regimen without consolidation transplant [2, 11]. In the present study, the hyper-CVAD regimen showed a significantly higher CR rate than CHOP and CHOP-based regimen (68 vs. 39%, *p* = 0.049). Thomas et al. [11] reported that the 3-year PFS was 66% in 33 adults after the hyper-CVAD regimen. Also, a large prospective study reported a 2-year DFS of 72.4% with a pediatric-like ALL chemotherapy, while post-remission allo-SCT (*n* = 17) did not bring any survival advantages [2]. However, patients included in the studies mentioned above were less aggressive and the limited cases of allo-SCT were hardly to make definitive conclusions. Although the results of intensive ALL-type regimen are very encouraging, the efficiency of chemotherapy is greatly compromised due to the severe hematological toxicity in Asians [12]. As a result, patients would benefit more from the upfront use of allo-SCT because of the shorten chemotherapy treatment duration and superior outcome brought with it.

The mediastinum is the most frequent site of presentation and recurrence, but the role of radiotherapy remains controversial. Our multivariate analysis suggested that radiotherapy associated with a statistically worse OS. Similarly, a large prospective study [13] found a better OS in the patients who did not received MedRad therapy (MRT) (*p* = 0.07), comparing with the prophylaxis MRT cohort (36 Gray). Considering the increasing risks of secondary disease and inferior survival, radiotherapy should be provided with caution.

To date, reliable prognostic factors have not been identified in LBL. Coleman et al. [14] proposed a risk statification for LBL patients in 1986. High-risk patients (defined as Ann Arbor stage IV disease with bone marrow or central nervous system involvement or initial elevated lactate dehydrogenase concentration) had a 5-year relapse-free survival rate of 19% compared with 94% for the low-risk patients (*p* = .0006). Despite that the Coleman system has been widely accepted, most further studies failed to identified these prognostic factors. Our risk factor analysis indicated poor ECOG-PS at diagnosis was an independent risk factor for both OS and PFS, which was in accordance with previous conclusions [2, 15]. One possible explanation was that higher tumor burden resulted in poorer performance status. Recently, novel risk factors such as molecular marker (NOTCH/FBXW7/RAS/PTEN) [2] have emerged and are waiting to be evaluated.

Our previous data demonstrated that allo-HSCT from suitable HRDs achieved similar outcome to MUDs and improved the outcome of high-risk leukemia [6]. In our current study, five patients received haploidentical grafts and three of them were in continuous remission. In view of the immediate availability of a HRD and promising clinical outcome, haplo-HSCT seems to be an alternative option for LBL.

In conclusion, this study underlines the superiority of allo-HSCT over conventional chemotherapy in adults with LBL in their first remission. More studies are needed to identify potential long-term toxicities and life quality after allogeneic hemopoietic stem cell transplantation.

## Electronic supplementary material

Supplementary Table 1

Supplementary Table 2

Supplementary Figure 1

Supplementary Figure 2
