# Supplementary material for: Allogeneic hematopoietic stem cell transplantation should be in preference to conventional chemotherapy as post-remission treatment for adults with lymphoblastic lymphoma
Source: Bone Marrow Transplant. 2018 Apr 30;53(10):1340–4. doi: 10.1038/s41409-018-0184-7 (PMC6173686; doi:10.1038/s41409-018-0184-7)
Supplement: Supplementary file 1 — Supplementary Table 1 [file 41409_2018_184_MOESM1_ESM.docx]

| Supplementary Table 1. Baseline characteristics. | | |
| --- | --- | --- |
| Variables | Number of Patients | % |
| N | 57 | 100 |
| Gender |  |  |
| Male | 37 | 65 |
| Female | 20 | 35 |
| Age years median (range) | 27(15-68) |  |
| Immunophenotye |  |  |
| T | 50 | 88 |
| B | 7 | 12 |
| Ann Arbor Stage |  |  |
| I | 3 | 5 |
| II | 3 | 5 |
| III | 9 | 16 |
| IV | 41 | 72 |
| N/A | 1 | 2 |
| B symptoms | 28 | 48 |
| ECOG performance status |  |  |
| 0 | 3 | 5 |
| 1 | 23 | 40 |
| 2 | 27 | 48 |
| 3 | 4 | 7 |
| Serum LDH level > normal | 23 | 40 |
| Medullary involvement | 29 | 51 |
| Mediastinal involvement | 30 | 53 |
| Pleural/pericardial affusion | 11 | 19 |
| Number of extranodal sites | |  |
| 0 | 12 | 21 |
| 1 | 23 | 40 |
| ≥2 | 18 | 31 |
| N/A | 5 | 8 |
| IPI Index |  |  |
| 0 or 1 | 17 | 30 |
| 2 | 9 | 16 |
| 3 | 15 | 26 |
| 4 or 5 | 10 | 18 |
| N/A | 6 | 10 |
| N/A, not available; ECOG, Eastern Cooperative Oncology Group; LDH, lactate dehydrogenase; IPI international prognosis index. | | |
