# Supplementary material for: Allogeneic hematopoietic stem cell transplantation should be in preference to conventional chemotherapy as post-remission treatment for adults with lymphoblastic lymphoma
Source: Bone Marrow Transplant. 2018 Apr 30;53(10):1340–4. doi: 10.1038/s41409-018-0184-7 (PMC6173686; doi:10.1038/s41409-018-0184-7)
Supplement: Supplementary file 2 — Supplementary Table 2 [file 41409_2018_184_MOESM2_ESM.docx]

| Supplementary Table 2. Induction Chemotherapy regimens | | |
| --- | --- | --- |
|  | Number of Patients | % |
| Hyper-CVAD A/B | 31 | 54 |
| CHOP and CHOP-based | 18 | 31 |
| VICP | 2 | 4 |
| VDLP | 3 | 5 |
| VDCP | 1 | 2 |
| VMCP | 1 | 2 |
| Ifosfamide and pharmorubicin | 1 | 2 |
| Hyper-CVAD A, cyclophosphamide, vincristine, doxorubicin, and dexamethasone; Hyper-CVAD B, methotrexate, cytarabine; CHOP, cyclophosphamide, doxorubicin, vincristine, prednisolone; VICP, vinsristine, daunorubicin, cyclophosphamide, prednisone; VDLP, vincristine, daunorubicin, L-asparaginase, prednisone; VDCP, vincristine, daunorubicin, cytarabine; VMCP, vinsristine, melphalan, cyclophosphamide, prednisone; COMEP, cyclophosphamide, vincristine, melphalan, etoposide, prednisone. | | |
