# Supplementary figures and images for: Allogeneic hematopoietic stem cell transplantation should be in preference to conventional chemotherapy as post-remission treatment for adults with lymphoblastic lymphoma
Source: Bone Marrow Transplant. 2018 Apr 30;53(10):1340–4. doi: 10.1038/s41409-018-0184-7 (PMC6173686; doi:10.1038/s41409-018-0184-7)

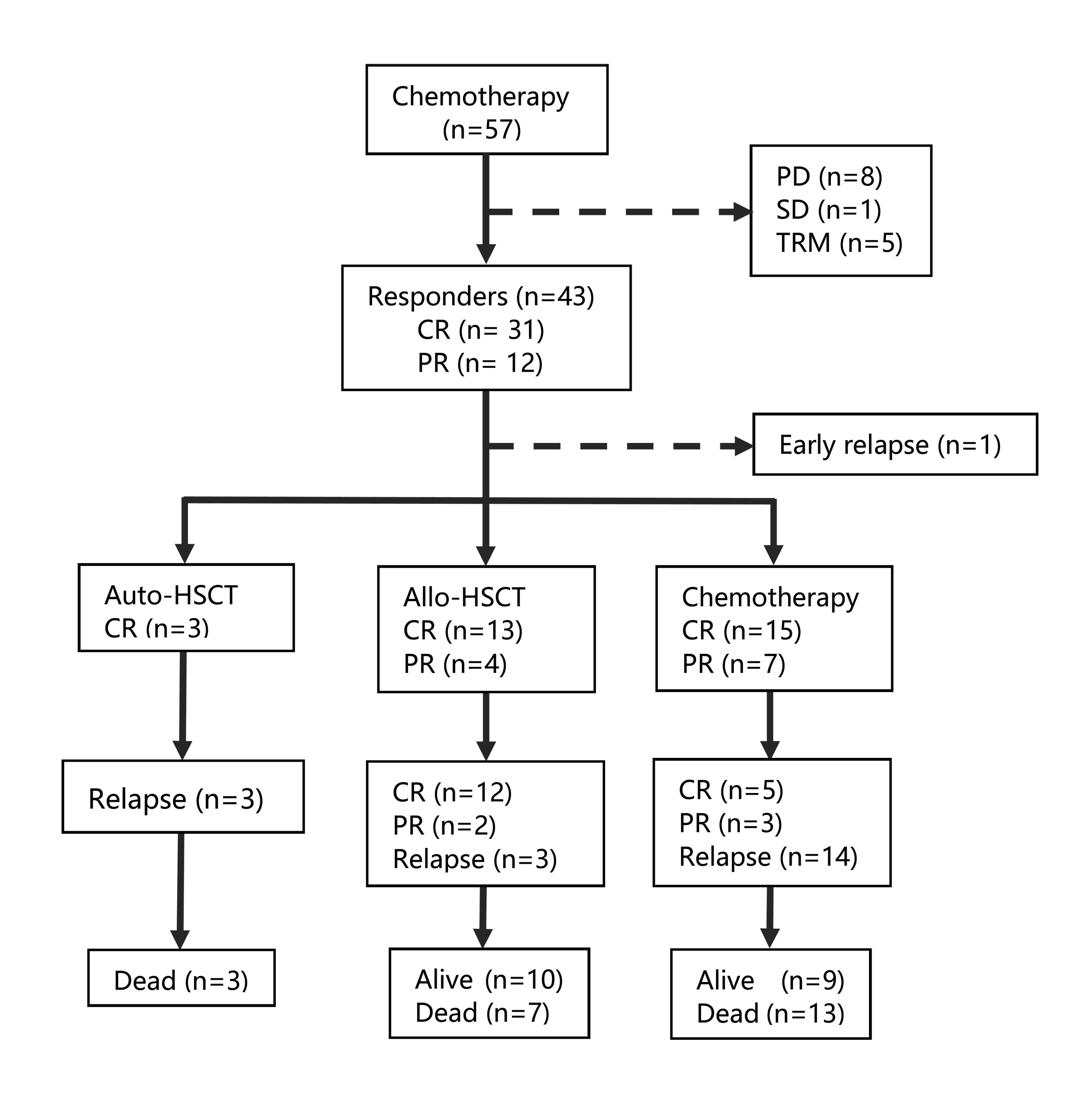

Supplement: Supplementary file 3 — Supplementary Figure 1 [file 41409_2018_184_MOESM3_ESM.tif]

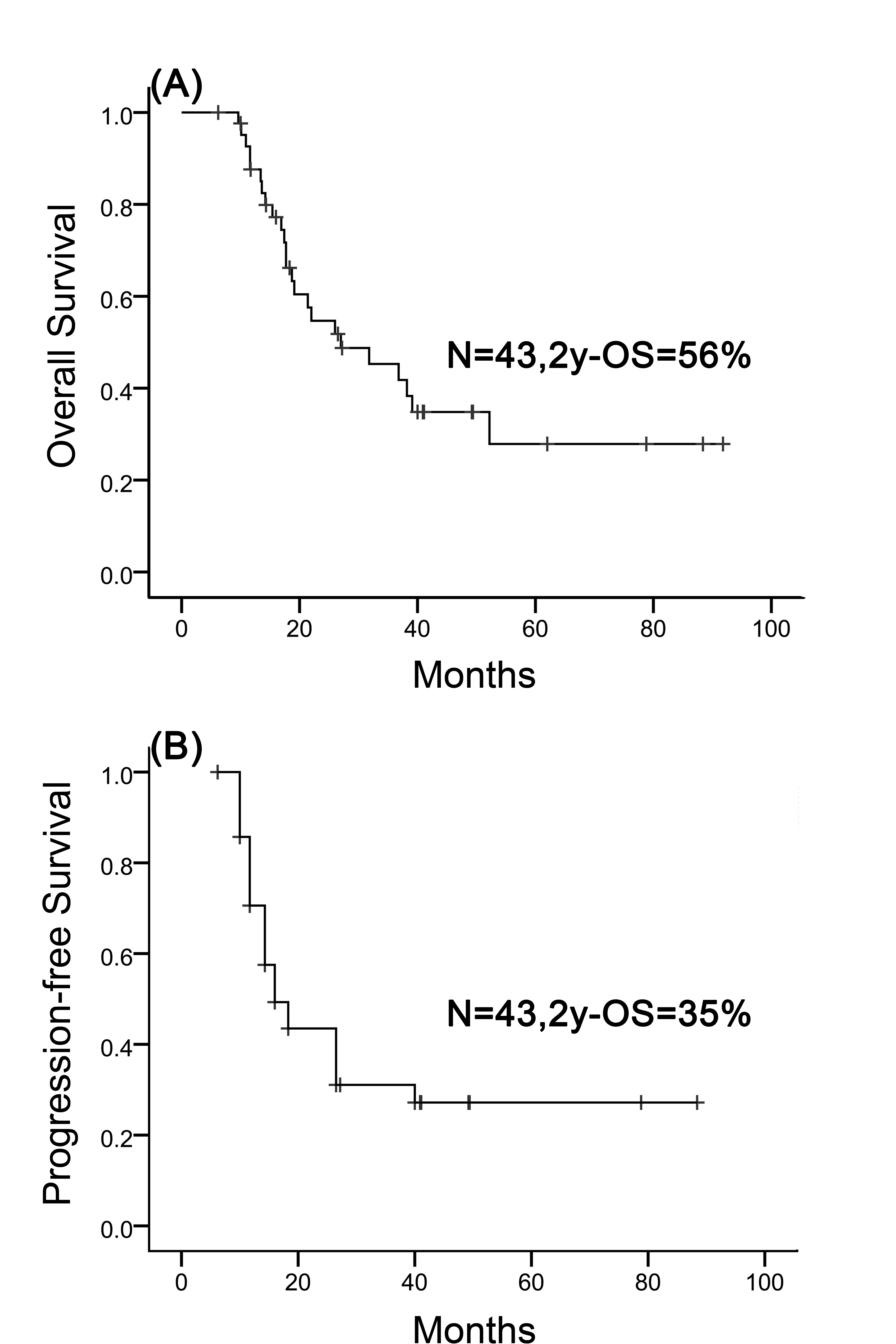

Supplement: Supplementary file 4 — Supplementary Figure 2 [file 41409_2018_184_MOESM4_ESM.tif]
